# The strengths and difficulties questionnaire as a screening instrument for norwegian child and adolescent mental health services, application of UK scoring algorithms

**DOI:** 10.1186/1753-2000-5-32

**Published:** 2011-10-12

**Authors:** Per Håkan Brøndbo, Børge Mathiassen, Monica Martinussen, Einar Heiervang, Mads Eriksen, Therese Fjeldmo Moe, Guri Sæther, Siv Kvernmo

**Affiliations:** 1 Department of Child and Adolescent Psychiatry, Divisions of Child and Adolescent Health, University Hospital of North-Norway, Tromsø, P.O. Box 19, 9038 Tromsø, Norway; 2 RKBU-North, Faculty of Health Sciences, University of Tromsø, 9037 Tromsø, Norway; 3 Institute of Clinical Medicine, University of Oslo, 0372 Oslo, Norway; 4 Alta Child and Adolescent Mental Health Service, Finnmark Hospital Trust, P.O. Box 1294, 9505 Alta, Norway; 5 School Psychology Services, Sørum Municipality, P.O.Box 113, 1921 Sørumsand, Norway; 6 Department of Adult Psychiatry, Division of General Psychiatry, University Hospital of North-Norway, Tromsø, P.O.Box 6124, 9291 Tromsø, Norway

## Abstract

**Background:**

The use of screening instruments can reduce waiting lists and increase treatment capacity. The aim of this study was to examine the usefulness of the Strengths and Difficulties Questionnaire (SDQ) with the original UK scoring algorithms, when used as a screening instrument to detect mental health disorders among patients in the Norwegian Child and Adolescent Mental Health Services (CAMHS) North Study.

**Methods:**

A total of 286 outpatients, aged 5 to 18 years, from the CAMHS North Study were assigned diagnoses based on a Development and Well-Being Assessment (DAWBA). The main diagnostic groups (emotional, hyperactivity, conduct and other disorders) were then compared to the SDQ scoring algorithms using two dichotomisation levels: 'possible' and 'probable' levels. Sensitivity, specificity, positive predictive value, negative predictive value, positive likelihood ratio, negative likelihood ratio, and diagnostic odds ratio (OR^D^) were calculated.

**Results:**

Sensitivity for the diagnostic categories included was 0.47-0.85 ('probable' dichotomisation level) and 0.81-1.00 ('possible' dichotomisation level). Specificity was 0.52-0.87 ('probable' level) and 0.24-0.58 ('possible' level). The discriminative ability, as measured by OR^D^, was in the interval for potentially useful tests for hyperactivity disorders and conduct disorders when dichotomised on the 'possible' level.

**Conclusions:**

The usefulness of the SDQ UK-based scoring algorithms in detecting mental health disorders among patients in the CAMHS North Study is only partly supported in the present study. They seem best suited to identify children and adolescents who do not require further psychiatric evaluation, although this as well is problematic from a clinical point of view.

## Background

A conservative prevalence estimate of psychiatric disorders in the Norwegian child and adolescent population (3-18 years old) is about 8% based on epidemiological surveys [1]. One large study showed a prevalence of 7% among children aged 8 to 10 years [2]. It is even more common for children and adolescents to suffer psychosocial impairment due to mental health problems, with an estimated 15 to 20% of this age group being affected [1]. Child and Adolescent Mental Health Services (CAMHS) in Norway are supposed to cover 5% of the child and adolescent population according to the Norwegian Health Authorities [3]. Service needs are not predicted solely by the number of children and adolescents diagnosed, but also by those who display psychosocial impairment without assigned diagnoses [4]. The gap between the prevalence/impairment estimates and CAMHS coverage highlights a very real capacity problem in the Norwegian mental health care system, which results in long waiting lists and added burdens for children and families who are in need of help. Similar capacity problems have been described in other countries [5,6]. Psychiatric screening procedures could help the situation by identifying whether a disorder is present, or if further evaluation is required [7]. The only way to achieve effective treatment is through accurate assessment. If less time is spent on the evaluation of healthy youngsters, and referrals to appropriate treatment programmes are more rapid, it could potentially increase treatment capacity, and decrease the long waiting lists in CAMHS.

The Strengths and Difficulties Questionnaire (SDQ), including the original UK scoring algorithms, is widely used as a screening tool for psychiatric disorders in clinical practice. It assesses child and adolescent behaviour, as well as the impact/impairment of any symptoms, based on information from parents, teachers and self-report [8,9]. Several studies, both international and from the Nordic countries, have reported that the psychometric properties of the SDQ are sound [10]. The accuracy measures of a screening test may vary due to the prevalence of a disorder and the population studied, and the majority of studies on the SDQ so far have taken place in population-based samples [11-17]. More limited studies have validated the diagnostic predictions rendered by the SDQ in clinical populations [5,18,19]. In just such a study by Goodman and colleagues [18], sensitivity ranged from 81% to 90%, and specificity from 47% to 84%. Positive predictive value (PPV) ranged from 35% (hyperactivity disorders) to 86% (emotional disorders) and negative predictive value (NPV) ranged from 83 to 98%. When replicating this study in an Australian CAMHS, Mathai and colleagues [5] reported a sensitivity that ranged from 36% (emotional disorders) to 93% (conduct disorders), or from 81 to 100% depending on the chosen dichotomisation. Hysing and colleagues [19] reported sensitivity (77%), specificity (85%), PPV (57%) and NPV (93%) for the SDQ among Norwegian children with chronic physical illnesses.

The aim of this study was to examine whether the application of specific scoring algorithms for the SDQ, as proposed by earlier findings from the UK [20], could be used for screening in order to detect mental health disorders among children and adolescents in the CAMHS North Study by examining sensitivity, specificity, PPV, NPV, positive likelihood ratio (LHR^+^), negative likelihood ratio (LHR^-^), and diagnostic odds ratio (OR^D^). To our knowledge, this is the first Norwegian study to examine the accuracy of the SDQ as a screening instrument for further evaluation in a clinical CAMHS sample.

## Methods

### Participants

All individuals aged 5 to 18 years, referred for diagnostic assessment to either the Child and Adolescent Mental Health Outpatient Clinic at the University Hospital of Northern Norway, or to the Alta Child and Adolescent Mental Health Outpatient Service at Finnmark Hospital Trust, by either a general practitioner or child welfare authorities, during the period September 2006 to December 2008 were invited by mail to participate (*N *= 1,032) in the CAMHS North Study. This study, carried out in the northern part of Norway evaluated clinical procedures, structures and treatment paths. The study included a broad spectrum of aims: to investigate factors that affect the waiting list, to evaluate examination and treatment time, to implement and validate structured instruments, and to investigate user satisfaction.

A total of 286 patients (28%) consented to participate in the CAMHS North Study, including 155 boys (54%) and 131 girls (46%) with a mean age of 11.11 years (*SD *= 3.35, *range *= 5-18 years). A total of 128 (45%) children were in the age range 5-10 years old (65% boys) and 158 (55%) adolescents were in the range 11-18 years (46% boys). Norwegian national statistics for CAMHS [20] shows a similar distribution for sex and age, with more boys (57%) than girls, and more adolescents (60% 13 years old or above) than children. Parents of participating patients provided information on their ethnicity, parental status, household income, socioeconomic stress, stress associated with work and work pressure, and stress associated with physical and mental health, which was recorded in the Development and Well-Being Assessment (DAWBA) background module (Table 1).

**Table 1:** Participant characteristics (*N *= 286) according to the DAWBA, Child and Adolescent Mental Health Services North Study, Norway, 2006-2008^a^

**Ethnicity**	Non-immigrant Norwegian	85%
	Sami people	3%
	Immigrant from Europe	4%
**Family (living with)**	Both biological parents	47%
	One biological parent	27%
	A biological parent and his/her new partner	13%
	Foster care	4%
**Household income**	Double income	56%
	One income	26%
**Socioeconomic stress**	No/minor	72%
	Major	14%
**Work/work pressure stress**	No/minor	63%
	Major	23%
**Physical/mental health stress**	No/minor	71%
	Major	15%

^a^Missing data for 8-18%.

Written informed consent was obtained before inclusion in the study. Parents gave consent for patients under 12 years of age. For patients between 12 and 16 years of age, written consents was obtained from both the parents and the patients. Patients over 16 years of age gave consent themselves according to Norwegian legislation. The Regional Committee for Medical Research Ethics and the Norwegian Social Science Data Services approved the study.

### Measures

The SDQ is a screening instrument that covers problems and resources relevant to the mental health and behaviour of children and adolescents aged 4 to 16 years [8]. It consists of three different versions: the parent version and teacher version rate behaviour for all ages; a self-reported version is used only among adolescents aged 11 to 16 years. The SDQ contains 25 items, covering five areas of clinical interest: hyperactivity/inattention (e.g. 'restless, overactive, cannot stay still for long'), emotional symptoms (e.g. 'many worries, often seems worried'), conduct problems (e.g. 'often has temper tantrums or hot temper'), peer relation problems (e.g. 'picked on or bullied by other children') and prosocial behaviour (e.g. 'kind to younger children'). The extended version of the SDQ, which is embedded in the DAWBA, also covers severity of difficulties, chronicity, overall distress, social and scholastic impairment, and burden to others (e.g. 'how long have these difficulties been present', 'do the difficulties upset or distress your child', 'do the difficulties interfere with your child's everyday life in the following areas') [9]. See http://www.sdqinfo.org for a full description of measure and items. Based on both symptoms and the corresponding impact reported by parents, teachers and self-report, predictive algorithms have been developed for a broad category, 'any disorder', as well as for three subcategories: conduct disorders, hyperactivity disorders, and emotional disorders. These algorithms, which are based on established British norms/cut-offs, have been tested in several cultures. They are described in detail by Goodman, Renfrew and Mullick [21] and syntaxes are available for download at http://www.sdqinfo.org, where normative data from different countries can be found. Country, gender and age affects the exact proportion, but these algorithms will classify approximately 80% of a population-based sample as 'unlikely' to have a psychiatric disorder, approximately 10% as 'possibly', and another 10% as 'probably' having a psychiatric disorder.

DAWBA was used to collect information both for clinically assigned diagnoses according to the International Classification of Diseases Revision 10 (ICD-10) and the Diagnostic and Statistical Manual of Mental Disorders, Fourth Edition (DSM-IV), and as the information source for the clinicians' severity ratings on the Health of the Nation Outcome Scales for Children and Adolescents, and the Children's Global Assessment Scale. The DAWBA interview is a package of measures of child and adolescent psychopathology for administration to multiple informants (parents, teachers, and/or self-response) who fill out the questionnaire electronically. The Norwegian version used in this study contains modules for diagnoses related to separation anxiety, specific phobias, social phobia, panic attacks and agoraphobia, post-traumatic stress disorder, generalised anxiety, compulsions and obsession, depression, deliberate self-harm, attention and activity, awkward and troublesome behaviour, developmental disorders, eating difficulties, and less common problems, as well as modules for background information and strengths. For each module there are both structured (yes/no) and semi-structured (free text) questions. Each module has screening questions, skip rules, and estimates of functional impairment. The DAWBA has shown good discriminative ability in both population-based samples and clinical samples, as well as across different categories of diagnoses [22]. Both in Norway and Great Britain, the DAWBA generates realistic estimates of prevalence for psychiatric illnesses as well as high predictive validity when used in public health services [2,23]. Good to excellent reliability between the rating clinicians has been reported in both British and Norwegian studies [2,24]. High levels of agreement between diagnoses assigned based on information solely from the DAWBA, and diagnoses based upon full clinical examination in addition to the DAWBA has been reported [25,26]

### Procedure

Four experienced clinicians (PHB, BM, EH, ME) independently assessed the patients included in the study (*N *= 286). The assessment was based on information collected from parents, teachers and/or self-report through the DAWBA, without face-to-face contact with the parents, teachers or patients themselves. The available information, including the SDQ, was identical for all four clinicians. To ensure there were enough cases for analysis, the diagnoses were separated into categories: emotional disorders (diagnoses related to separation anxiety, specific phobias, social phobia, panic attacks and agoraphobia, post-traumatic stress disorder, generalised anxiety, compulsions and obsession, depression, and deliberate self-harm), hyperactivity disorders (diagnoses related to attention and hyperactivity), conduct disorders (diagnoses related to awkward and troublesome behaviour), and other disorders (diagnoses related to developmental disorders, eating difficulties, and less common problems). Comorbidity was registered whenever the diagnostic criteria for more than one diagnosis were met, without attention to the exclusion rules of the ICD-10.

The first 100 patients were assigned diagnoses by four independent clinicians, and consensus diagnoses were assigned for cases with disagreement between the clinicians (Brøndbo, Mathiassen, Martinussen, Heiervang, Eriksen, Kvernmo: Rater Agreement for Diagnoses and Severity of Mental Health Problems in a Naturalistic Clinical Setting, submitted). As good agreement was found between the clinicians' diagnoses and consensus diagnoses in these first 100 cases, (*κ *= 0.70-1.00), the remaining 186 patients were divided and diagnosed by only one of the four clinicians. Only cases with diagnostic ambiguity were discussed (*N *= 14). Previous studies, such as the British Child and Adolescent Mental Health Survey 1999 [23,24] and the Bergen Child Study [2] have used similar procedures.

### Statistical analyses

All statistical analyses were performed using SPSS version 16. Chi-square analyses were conducted to compare findings for children and adolescents, both for levels of SDQ dichotomisation and for the DAWBA diagnoses. For the calculation of screening efficiency in terms of sensitivity, specificity, PPV, NPV, LHR^+^, LHR^-^, and OR^D^, results were dichotomised on the original probability categories in the SDQ scoring algorithm (unlikely, possible, and probable). In a first instance calculations were made where the categories unlikely and possible were labelled 'test negative' and the third category probable was labelled 'test positive' (hereafter referred to as 'probable' dichotomisation level). In the second calculation only the category unlikely was labelled 'test negative' and the second and third categories possible and probable were labelled 'test positive' (hereafter referred to as the 'possible' dichotomisation level). Applying the 'probable' dichotomisation level will classify approximately 90% of a population-based sample as having a negative test, whereas the 'possible' dichotomisation level will yield a result of 'test negative' for approximately 80% of the same sample.

Sensitivity and specificity are one way of quantifying the diagnostic accuracy of a test [27]. Sensitivity is the ability of the screening instrument to generate a true positive result for someone with the diagnostic category of interest. Specificity is the ability of the instrument to generate a true negative result for someone without the diagnostic category of interest [28]. The design used is outlined in Table 2. To calculate sensitivity and specificity the following equations were used: *sensitivity = a/(a + c), specificity = d/(b + d)*.

**Table 2:** Performance of a screening test

**SDQ**	**Gold standard**			
	
		**Diagnosis**	**No diagnosis**	**Total**
		
	Test positive	*a*	*b*	*a + b*
	Test negative	*c*	*d*	*c + d*
		
	Total	*a + c*	*b + d*	*a + b + c + d*

*Note*. a = True positive, b = False positive, c = False negative, d = True negative.

Sensitivity and specificity are important to determine diagnostic accuracy, but they are not useful in estimating the probability of a disorder [29]. PPV and NPV refer to the probability that a positive or negative test result reflects the correct diagnosis [28]. These values vary according to the prevalence of a disorder in a given population [7]. For example PPV for a disorder with low prevalence can be low even if the sensitivity and specificity are high. To calculate PPV and NPV the following equations were used: *PPV = a/(a + b), NPV = d/(c + d) *(Table 2).

LHRs are ratios of probabilities, and are used to summarise diagnostic accuracy on the basis of sensitivity and specificity [30]. The LHR provides information on how a positive or negative test result changes the likelihood of a person to have a certain diagnosis. To calculate LHR^+ ^and LHR^- ^the following equations were used: *LHR^+ ^= sensitivity/(1 - specificity), LHR^- ^= (1 - sensitivity)/specificity*.

A single measure that summarises the discriminative ability of a test is the OR^D^, which is computed by the following equation: *LHR^+ ^/LHR^-^*. The OR^D ^is relatively independent of changes in both spectrum and prevalence, and therefore is a robust measure for dichotomised results. For clinical purpose 'acceptable' accuracy will vary depending on the aim (i.e. to confirm the absence or presence of a disorder) and due to the consequences for the patient. The LHR^+^, the LHR^-^, and the OR^D ^were interpreted according to the rule of thumb described in Fischer, Bachmann and Jaeschke [31], where potentially useful tests (i.e. may alter clinical decisions) usually are characterised by LHR^+ ^greater than 7 or LHR^- ^less than 0.3, or an OR^D ^above 20.

## Results

For all patients (*N *= 286) clinician-assigned diagnoses were recorded based on information collected from parents, teachers and/or self-report through the DAWBA, also including the SDQ [32]. The corresponding questionnaire was completed by 93% of parents, 72% of teachers, and 84% of adolescents 11 years or older (*N *= 158). Multiple versions of the DAWBA were completed for 87% of patients. Only 13% of patients had a single version of the DAWBA completed: either the parent version (10%) or the self-report (3%). A total of 66% of patients were assigned a psychiatric diagnosis based on the DAWBA, and of those almost one-third (21%) were assigned comorbid diagnoses. A diagnosis of emotional disorder was assigned to 34% of patients, and two out of three had this as their only diagnosis. A diagnosis of hyperactivity disorder was assigned to 18% of patients, and more than two out of three also had one or more comorbid diagnoses. Conduct disorder diagnoses were assigned to 31% of patients and about half of them also had one or more comorbid diagnoses. Other diagnoses were assigned to 7% of the patients and nine out of 10 also had one or more comorbid diagnoses. The most common comorbid diagnoses were hyperactivity disorder in combination with conduct disorder (10%) and emotional disorder in combination with conduct disorder (8%). A total of 2% were assigned diagnoses from more than two categories ('emotional', 'hyperactivity', 'conduct', 'other').

Table 3 presents the SDQ-predicted diagnoses for both dichotomisation levels and DAWBA diagnoses, i.e., the 'gold standard' based on the diagnoses assigned by the four clinicians. As expected, the amount of SDQ-predicted diagnoses was highest when the 'possible' dichotomisation level was applied for all disorders. For the prevalence of 'any disorder', the 'possible' dichotomisation level was 89%, compared to 72% for the 'probable' dichotomisation level, and 66% for the DAWBA diagnoses. In addition, the rates of SDQ-predicted diagnoses using the 'probable' dichotomisation level were higher than the rates of DAWBA diagnoses for all categories except emotional disorders. As expected, there were significant differences between children and adolescents in terms of diagnoses, with more of 'any disorder', more emotional disorders and less hyperactivity disorders in adolescents (11-18 years), compared to children (5-10 years).

**Table 3:** SDQ Predicted Diagnoses and Clinical DAWBA Diagnoses among 286 patients in the Child and Adolescent Mental Health Services North Study, Norway, 2006-2008

	SDQ - 'possible'				SDQ - 'probable'				DAWBA diagnoses			
	**All ages^a ^**	**Child^b^**	**Youth^c^**	**χ^2^**	**All ages^a^**	**Child^b^**	**Youth^c^**	**χ^2^**	**All ages^a^**	**Child^b^**	**Youth^c^**	**χ^2^**
	
**Any disorder**	255 (89%)	117 (91%)	138 (87%)	1.21	207 (72%)	94 (73%)	113 (72%)	0.13	188 (66%)	76 (59%)	112 (71%)	4.16*
**Emotional disorders**	164 (57%)	61 (50%)	103 (65%)	8.89**	70 (25%)	19 (15%)	51 (32%)	11.63**	98 (34%)	24 (19%)	74 (47%)	24.76**
**Hyperactivity disorders**	181 (63%)	87 (68%)	94 (60%)	2.19	85 (30%)	50 (39%)	35 (22%)	9.68**	51 (18%)	33 (26%)	18 (11%)	9.99**
**Conduct disorders**	168 (59%)	83 (65%)	85 (54%)	3.56	123 (40%)	60 (47%)	63 (40%)	1.41	88 (31%)	45 (35%)	43 (27%)	2.09
**Comorbidity**	176 (62%)	78 (61%)	98 (62%)	0.04	62 (22%)	30 (23%)	32 (20%)	0.42	59 (21%)	28 (22%)	31 (20%)	0.22

^a ^All ages = 5-18 years, ^b^Child = 5-10 years, ^c^Youth = 11-18 years

* p < 0.05

** p < 0.01

Table 4 presents the screening efficiency of the SDQ in terms of sensitivity, specificity, PPV, NPV, LHR^+^, LHR^-^, and OR^D ^for the different diagnostic categories of emotional disorders, hyperactive disorders and conduct disorders, as well as 'any disorder'. When the 'probable' dichotomisation level was applied, none of the LHR^+ ^results were in the interval for potentially useful tests That means that the likelihood of a person having a diagnosis after a positive test is between 1.78 to 3.91 times bigger, which is not enough to be interpreted as having a potential to alter clinical decisions. The categories hyperactive disorders, conduct disorders, and 'any disorder' were all in the LHR^- ^interval for potentially useful tests. That means that the likelihood of a person having one of those diagnoses after a negative test is between 0.23 to 0.29 times smaller, which is enough to be interpreted as having a potential to alter clinical decisions. None of the OR^D ^results were in the interval for potentially useful tests as indicated by the guidelines provided by Fischer, Bachmann and Jaeschke [31]. After applying the 'possible' dichotomisation level, none of the LHR^+ ^results (1.25-2.30) were in the interval for potentially useful tests. The categories hyperactive disorders, conduct disorders, and 'any disorder' were all in the LHR^- ^interval for potentially useful tests, i.e. the likelihood of a person having 'any disorder' after a negative test is 0.18 times smaller and the likelihood of hyperactivity or conduct disorder after a negative test is even smaller (0.00-0.06). Likewise, the OR^D ^results for hyperactive disorders and conduct disorders were in the interval for potentially useful tests. This means that the chances of a conduct or hyperactivity disorder with a positive test is 39.26 times, respectively infinitely, bigger than the occurrence of those disorders with a negative test, which is enough to be interpreted as a result of discriminative ability with potential to alter clinical decisions.

**Table 4:** Screening Efficiency for the Diagnostic Categories for Different Levels of Dichotomisation among 286 patients in the Child and Adolescent Mental Health Services North Study, Norway, 2006-2008

	Sensitivity **(prob**^a^**/poss**^b^**)**	Specificity **(prob**^a^**/poss**^b^**)**	**PPV (prob**^a^**/poss**^b^**)**	NPV **(prob**^a^**/poss**^b^**)**	LHR^+^ **(prob**^a^**/poss**^b^**)**	LHR^-^ **(prob**^a^**/poss**^b^**)**	OR^D^ **(prob**^a ^**95% CI)**	OR^D^ **(poss**^b ^**95% CI)**
**Emotional disorders**	0.47/0.81	0.87/0.55	0.66/0.48	0.76/0.84	3.68/1.78	0.61/0.45	6.05 (3.37-10.84)	5.04 (2.83-8.98)
**Hyperactivity disorders**	0.77/1.00	0.80/0.45	0.46/0.28	0.94/1.00	3.91/1.81	**0.29/0.00**	13.35 (6.48-27.51)	**^c^**
**Conduct disorders**	0.83/0.97	0.75/0.58	0.59/0.51	0.91/0.98	3.29/2.30	**0.23/0.06**	14.41 (7.59-27.36)	**39.26 **(12.00-128.46)
**Any disorder**	0.85/0.96	0.52/0.24	0.77/0.71	0.65/0.74	1.78/1.25	**0.29/0.18**	6.20 (3.53-10.90)	6.90 (2.95-16.12)

^a^Dichotimised on probable level ('unlikely and 'possible' labelled 'no diagnosis', 'probably' labelled 'diagnoses'), ^b^Dichotomised on possible level (unlikely labelled 'no diagnosis', 'possible' and 'probably' labelled 'diagnoses'), ^c^Not possible to calculate due to zero in the denominator. Categorised as potentially useful.

*Note*. Potentially useful tests as indicated by the guideline provided by Fischer, Bachmann and Jaeschke [20] in bold.

## Discussion

The aim of the study was to examine the usefulness of the application of specific scoring algorithms for the SDQ, as proposed by earlier UK findings, when used as a screening test to detect mental health disorders among patients in the CAMHS North Study. Sensitivity and specificity are important to clinicians because these measures indicate how many people with disorders the SDQ can correctly identify. Our results varied according to the dichotomisation level applied in the SDQ diagnostic algorithm, and also varied by diagnostic category.

For both levels of dichotomisation, emotional disorders had the lowest sensitivity. Our results for the most commonly used 'probable' dichotomisation level, which yielded a cut-off of approximately 90% in epidemiological samples, were almost identical to those reported by Mathai and colleagues [5]. Goodman and colleagues [21] also reported a lower sensitivity for emotional disorders than for the other diagnostic categories in the British sample, but not as low as in the present study. This difference may be an effect of Norwegian parents' and teachers' 'blind spot', or 'normalising' view for emotional difficulties, which was also reported by Heiervang, Goodman and Goodman [33]. Given that the parents describe emotional difficulties in the semi-structured questions (free text) without reporting the same difficulties as problematic in the structured (yes/no) part, this may explain why the rates of clinician assigned DAWBA diagnoses are higher than the SDQ 'probable' screening rate for emotional disorders. This is in contrast to all other categories of disorders where the rates of clinician assigned DAWBA diagnoses are the lowest ones as expected, as a consequence of the screening cut-offs set at approximately 80% and 90% respectively, chosen to ensure inclusion of most cases in a population with a prevalence of psychiatric disorders of 7-8%. It is also generally accepted that parents are insensitive to children's emotional symptoms and that adolescents' reports of emotional problems are more valid than their parents' and teachers' reports [34,35]. This knowledge may have affected the assessments of the diagnosing clinicians in our study, and resulted in lower sensitivity. For both hyperactivity and conduct disorders, as well as for 'any disorder', our results showed high sensitivity, ranging from 77% to 100%, Nevertheless, these values were lower than those reported by Goodman and colleagues [21] for hyperactivity and conduct disorders in their English sample, and for hyperactivity disorders in their Bangladeshi sample. Compared to Mathai and colleagues [5], our results were substantially more sensitive for hyperactivity disorders, and a little less sensitive for conduct disorders and emotional disorders. As expected, our results for the 'possible' dichotomisation level, which yielded a cut-off at approximately 80%, were more sensitive for psychiatric disorders.

Specificity was also dependent on dichotomisation level and diagnostic category. All specificity results for the 'possible' dichotomisation level were lower than those for the 'probable' dichotomisation level. The specificity for 'any disorder' was the lowest, regardless of the level of dichotomisation and considerably lower than the specificity for the other individual categories. All specificity results were comparable to those reported by Goodman and colleagues [21], except for conduct disorders, for which specificity was substantially higher than in the British sample. This may be due to differences between the countries, in that the degree of reporting problems in Great Britain may be higher, whereas Norwegian parents and teachers tend to report fewer problems. In contrast to emotional disorders, the lower SDQ questionnaire scores for conduct problems seems to reflect a real and substantial lower prevalence of conduct disorders in Norway compared to Great Britain [33]. The above-mentioned studies did not report screening efficiency statistics for the diagnostic category 'any disorder'. Overall our sensitivity and specificity results strengthen the earlier reported usefulness of the SDQ as a screening instrument for mental health problems when used in epidemiological research. Regarding clinical use, despite differences in culture and language, the scoring algorithms worked equally well in the Norwegian CAMHS North Study as in English, Bangladeshi, and Australian clinics. With the most common cut-off at approximately 90%, the SDQ will correctly identify four out of five children with psychiatric diagnoses, except for emotional disorders, and also correctly identify most children without diagnoses, except for 'any disorder'. Unfortunately 23 to 54% of these diagnoses will be false positives and 6 to 35% of negative screening results will be false negatives, depending on the category of diagnoses. On the other hand, a cut-off point at approximately 80% will correctly classify almost all children with one or more diagnoses, but only half or less of children with negative screening results will be correctly classified. The range of false positives will increase to between 29 and 72% and the false negatives decrease to between 0 and 26%, depending on the category of diagnoses. Choice of cut-offs may depend on the relative importance of false positives and false negatives, respectively. For research purposes both scenarios are sufficient, but not for clinical purposes, for which the rates of false positives are not acceptable.

Sensitivity and specificity are important from a population perspective, but for patients and their clinicians PPV, NPV, LHR^+^, LHR^- ^and OR^D ^may be more informative, as they show the probability of a disorder, given a positive or negative screening result. Compared to the findings from a Norwegian study of children with chronic physical illnesses [19], our results showed a higher PPV, but a lower NPV for 'any disorder'. Our results by diagnostic category, showed a high NPV and lower PPV, which were very similar to the results reported by Goodman and colleagues [21]. This indicates that the SDQ functions considerably better as a tool to rule out, rather than to confirm, possible psychiatric diagnoses. The pattern may be even more significant when mental health problems are combined with chronic physical illness.

To our knowledge LHR^+/- ^and OR^D ^have not been reported in previous studies. Our results showed that when using the most common dichotomisation ('probable' level) at approximately 90%, none of the diagnostic categories are in the OR^D ^interval for potentially useful tests. This may seem strange since relative high OR^D^'s were reported (i.e. 6.05-14.41), but is mainly explained by too wide confidence intervals to consider the OR^D^'s as stable high estimates. However hyperactivity disorders, conduct disorders, and 'any disorders' are in the LHR^- ^interval for potentially useful tests. When the 'possible' dichotomisation level was used all LHR^+ ^results were worse and all LHR^- ^results were better, yielding OR^D ^results in the interval for potentially useful tests for diagnostic categories of hyperactivity disorder and conduct disorder. For a patient with a negative screening result this is good news, because it means that this result is almost certainly correct. However, for a clinician, and for patients with positive screening results, it is also important that the PPV and LHR^+ ^are high in order to reduce both economic and emotional costs associated with unnecessary further evaluations of patients that are not afflicted with the disorder of interest.

The clinical implication of our results is that the SDQ by itself is not a sufficient screening instrument for psychiatric disorders when used among patients in the CAMHS North Study in Norway. Our results showed that the SDQ could be better utilised to detect the presence of 'any' diagnoses, rather than more specific diagnostic categories. On the contrary, the SDQ is better at ruling out the presence of specific categories of psychiatric disorders than ruling out the actual presence of 'any disorder'. Our results are in accordance with previous studies [5,19,21] that clearly showed the unsuitability of SDQ for diagnostic purposes in a clinical setting, but contrary to these studies our results call into question the usefulness of SDQ to identify children who are in need of further psychiatric evaluation, as PPV and LHR^+ ^results are low. According to our results the SDQ is best used to identify those children and adolescents who do not need further psychiatric evaluation. Such clinical practice is however problematic since children suffering from monosymptomatic disorders (e.g. tic disorders, enuresis, eating disorders) not will be identified with screening with the SDQ.

There are some limitations to this study. One is that the diagnosing clinicians were not blinded to the SDQ predictions while assigning the clinical diagnoses based on the DAWBA. This might have affected the clinical assessment and biased the results towards better agreement between the SDQ and the clinical diagnoses. Some previous studies have blinded the clinical experts to avoid this bias [5,21], although others [19] have used the same procedure reported in the present study. Another bias towards better agreement is that both SDQ information and DAWBA information were collected at the same time, which prevents changes in mental health status between assessments. On the other hand, multiple informants as in our study are often a clinical necessity, but from a research point of view this more complex and sometimes contradictory information may weaken the agreement between raters. The strength of our procedure lies in its ecological validity, as our diagnostic procedure is quite similar to the ordinary day-to-day practise, including the use of the original UK scoring algorithms, in Norwegian CAMHS.

Another limitation is the assumption of the clinician consensus diagnoses as the gold standard. As previously documented, there is poor agreement between structured interviews and clinicians' assigned diagnoses, and little knowledge about the most valid methods [36]. There is no single objective feature that distinguishes any mental health diagnosis. Costello, Egger, and Angold [37] stated that structured interviews are the closest we can come to a gold standard for psychiatric diagnoses. Thus, the assignment of clinical experts aided by a structured interview such as the DAWBA may be considered the best available reference for comparison. Such procedures are imperfect, but nevertheless valuable as long as mental health diagnostics are based on developmental history, behavioural observations and reported difficulties in everyday life.

Further research is needed to find out if combining the SDQ with other measures of symptoms and severity can improve the ability to detect mental health disorders among patients referred to CAMHS. Also more efficient case-finding strategies, as suggested by Ullebø et. al. for ADHD phenotype [38], can optimize the potential of SDQ as a screening instrument for Norwegian CAMHS. Another aspect that merits further research is the identification of certain characteristics of either the patient or the other SDQ informants that might enhance the risk of false-positive or false-negative results. With a future database, large enough to subdivide the overall sample, subgroup-specific algorithms could be established and reported to facilitate comparisons between different clinical samples (e.g. with respect to age, gender, diagnostic categories) as well as identification of protective and/or risk factors.

## Conclusions

In conclusion, the ability of the SDQ to detect mental health disorders among patients referred to CAMHS is not sufficient for clinical purposes. When used as a screening instrument to determine whether further evaluation is warranted in a clinical CAMHS sample the SDQ seems best suited to identify children and adolescents who do not require further psychiatric evaluation, although this as well is problematic from a clinical point of view.

## List of abbreviations

CAMHS: Child and Adolescent Mental Health Services; DAWBA: Development and Well-Being Assessment; DSM-IV: Diagnostic and Statistical Manual of Mental Disorders, Fourth Edition; ICD-10: International Classification of Diseases Revision 10; LHR^-^: Negative likelihood ratio; LHR^+^: Positive likelihood ratio; NPV: Negative predictive value; OR^D^: Diagnostic odds ratio; PPV: Positive predictive value; SDQ: Strengths and Difficulties Questionnaire.

## Competing interests

PHP, BM and SK provide teaching to clinics on the use of the SDQ and DAWBA. EH is the director and owner of Careahead, which provides teaching and supervision services to clinics on the use of the SDQ and DAWBA.

## Authors' contributions

PHB was responsible for the rating data, data analysis and manuscript writing. BM participated in the rating of data, data analysis and commented on the written drafts. MM supervised the writing and commented on the written drafts. EH and ME participated in the rating of data and commented on the written drafts. TFM and GS participated in the manuscript writing and commented on the written drafts. SK designed and coordinated the study, supervised the manuscript writing and commented on the written drafts. All authors read and approved the final manuscript.
